# Integration of Error Compensation of Coordinate Measuring Machines into Feature Measurement: Part II—Experimental Implementation

**DOI:** 10.3390/s16101705

**Published:** 2016-10-14

**Authors:** Roque Calvo, Roberto D’Amato, Emilio Gómez, Rosario Domingo

**Affiliations:** 1 Department of Mechanical Engineering, Chemistry and Industrial Design, Universidad Politécnica de Madrid, Ronda de Valencia 3, Madrid 28012, Spain; r.damato@upm.es (R.D.); emilio.gomez@upm.es (E.G.); 2 Department of Construction and Manufacturing Engineering, Universidad Nacional de Educación a Distancia (UNED), C/Juan del Rosal 12, Madrid 28040, Spain; rdomingo@ind.uned.es

**Keywords:** CMM uncertainty, CMM error mapping, CMM verification, flatness measurement, angle measurement, circularity measurement

## Abstract

Coordinate measuring machines (CMM) are main instruments of measurement in laboratories and in industrial quality control. A compensation error model has been formulated (Part I). It integrates error and uncertainty in the feature measurement model. Experimental implementation for the verification of this model is carried out based on the direct testing on a moving bridge CMM. The regression results by axis are quantified and compared to CMM indication with respect to the assigned values of the measurand. Next, testing of selected measurements of length, flatness, dihedral angle, and roundness features are accomplished. The measurement of calibrated gauge blocks for length or angle, flatness verification of the CMM granite table and roundness of a precision glass hemisphere are presented under a setup of repeatability conditions. The results are analysed and compared with alternative methods of estimation. The overall performance of the model is endorsed through experimental verification, as well as the practical use and the model capability to contribute in the improvement of current standard CMM measuring capabilities.

## 1. Introduction

The needs of quality control demand tight standards of product inspection and the development a reliable approach for the measurement of manufacturing parts supported by the industrial metrology. Coordinate Measuring Machines (CMM) are versatile instruments used for precision inspection in industrial verification rooms or in research laboratories. Capable of measuring complex parts, a coordinate-measuring machine is a measuring system involving several steps. Firstly, the data extraction of the spatial coordinates of points belonging to the surfaces of an artefact through a probe, a complex sensor itself. Next, processing of the gathered data. Finally, the generation of geometry substitution from fitting algorithms and its use for measurement result. A new model of CMM error compensation by axis and the associated uncertainty estimation have been developed in [1].

An overall CMM error bounding is currently a standard approach for machine verification to complement the best value of measurement. It is a black box treatment of machine bounds of acceptance or verification by ISO 10360-2 standard. CMM’s acceptance or verification tests are useful for performance verification and contractual agreement on CMM performance, but they are outside the ordinary metrology chain of measurement traceability. Nevertheless, international standards do not renounce to uncertainty estimation in CMM measurements, but they consider firstly the error, while uncertainty should be also considered, in particular to comply with ISO/IEC 17025 [2]. Complementing the ISO 10360-2 [3] verification test, the test uncertainty can be estimated by ISO 23165 [4]. In practical terms of CMM use, the indication can be bounded by the maximum permissible error in the rated operating conditions, or for particular measurements ISO 15530-3 [5] can be used to evaluate measurement uncertainty under strict procedures.

The aim of this study is to propose a new compensation error model for CMM measurements based on the experimental behavior of the CMM, including the estimation of the associated uncertainty. This work is presented in two parts. In Part I [1] the basic error model of length was developed by introducing basic considerations of CMM measurement. A linear model of error by axis and its aggregation was proposed. Part I of this work included and interpreted the models for flatness, angle and dihedral angle measurement and roundness measurement. In Part II, the formulations obtained in Part I of the paper are used to develop the experimental implementation. The experimental error mapping from standard verification test of a moving bridge CMM is included in Section 2. The overall interpretation of the model from experimental results is evaluated in Section 3. Next, in Section 4 the measurement model is implemented to evaluate the length and angle from calibrated artefacts (gauge blocks), the roundness from a precision glass hemisphere and the flatness of the CMM granite. Results are discussed and the overall performance of the model and its experimental validation and use are analysed with concluding remarks.

## 2. CMM Experimental Error Mapping

### 2.1. The Mapping of Error by Axis

A Moving Bridge CMM TESA Micro-Hite 3D is used to evaluate experimentally its errors. Its field of measurement is XxYxZ of 450 mm × 500 mm × 460 mm and it has a resolution of 1 μm. According to ISO 10360-2, calibrated artefacts for bidirectional length measurements are used, in particular a set of calibrated gauge blocks of grade 0 up to 100 mm nominal and grade 2 from 125 mm nominal onwards. The uncertainty of those gages is below the resolution of the CMM, so that the deviations from the calibrated length will be assigned to measurement error. Measuring is developed under controlled and stable temperature at 20 ± 1 °C and the tests have been carried out by the same skilled member of our team. The CMM is set up according to the manufacturer’s instructions, with the standard approach of performing the measurement after compensating for any known bias.

**Figure 1:**
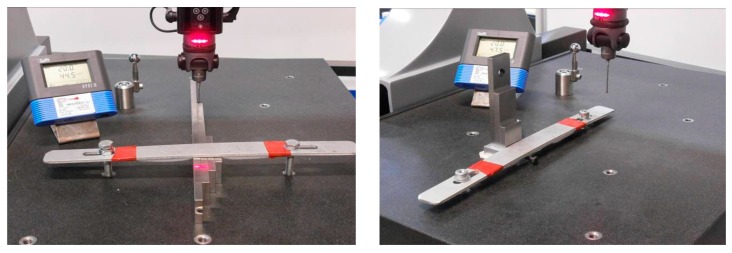
CMM verification by measuring a calibrated gauge step set.

The test includes five different gauge blocks from 50 mm to 300 mm nominal that cover at least 66% of the CMM field of measurement of every axis. Gauges are positioned at the middle of the volume of the CMM (Figure 1). A set of five points on each block side in a bidirectional measurement is followed for all three axes X, Y and Z, with five repetitions. A total of 50 measurements per gauge block grouped into five series of bilateral measurements is taken. This grouping follows the gauge block calibration certificate, where the assigned value of the block is obtained from five measured points on a face, with reference to the opposite face. In the case of Z axis, standing on the granite table, the second reference face in the bilateral measurement technique is the granite table itself. Ordinary ISO verification test only requires three repetitions for repeatability verification. The study intends to take advantage of the standard verification techniques with a different purpose of evaluating the mean error and its variability on the three main axis positions included in the model proposed in Part I.

The measurements are obtained through a Renishaw probe of the minimum length compatible with the step block measurement and choosing the probe of maximum tip diameter following the good practices and CMM manufacturer recommendations.

**Figure 2:**
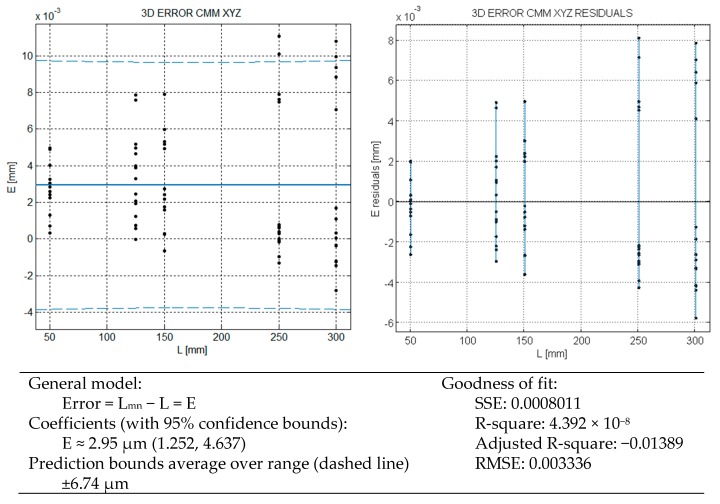
CMM bulk error model.

The aggregated results of the verification of calibrated blocks aligned with each axis are shown in Figure 2. The overall linear regression model yields an average error E of 2.95 µm. Even when the verification only includes the three main axes the overall error result E_MPE,L_ of 6.7 µm (prediction bounds at 95% confidence) is in close agreement with the verification of the machine at the time of acquisition (±6.7 µm over 300 mm) and a previous recent machine verification [6], both following direct application of the standard verification test ISO 10360-2. This ISO test evaluates an overall model of maximum permissible error in the rated operating conditions of the machine and without distinction of error by each axis.

In the regression model, the length of the gauge blocks is the known variable and the error is the random variable. The residual graph shows a spread growing with the length of measurement, so the basic assumption of the regression model of uniform variance across the known variable (homocedastic) is violated. The ISO verification test assumes the random error spread can grow by the length of measurement (heterocedactic) when proposes E_MPE,L_ = A + BL as one of the possible models of error for the machine. Even when nothing is indicated in the ISO 10360-2 standard, this weakness of using the regression technique could be eventually overcome by weighting the measurement with the inverse of their variance [7]. In fact this behavior of uncertainty growing with length is also the basic model of uncertainty of calibrated gauge block sets. The growing spread with the length can be the sign of an aggregated behavior that ordinary ISO verification test does not separate by grouping the results of the three axes.

**Figure 3:**
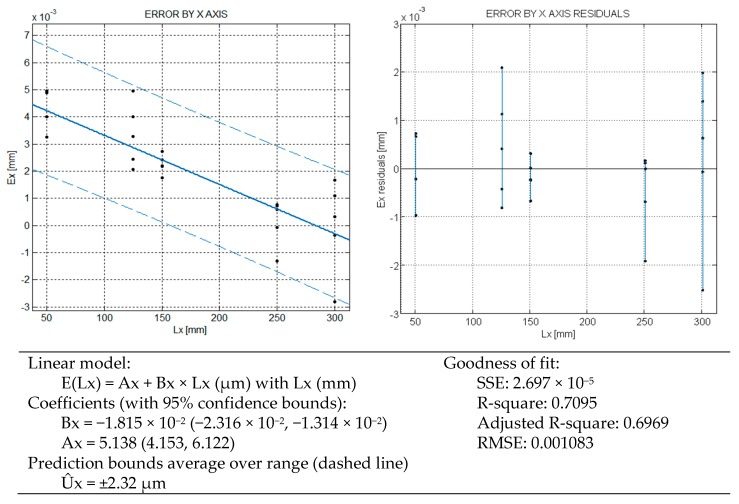
CMM error model by X axis.

**Figure 4:**
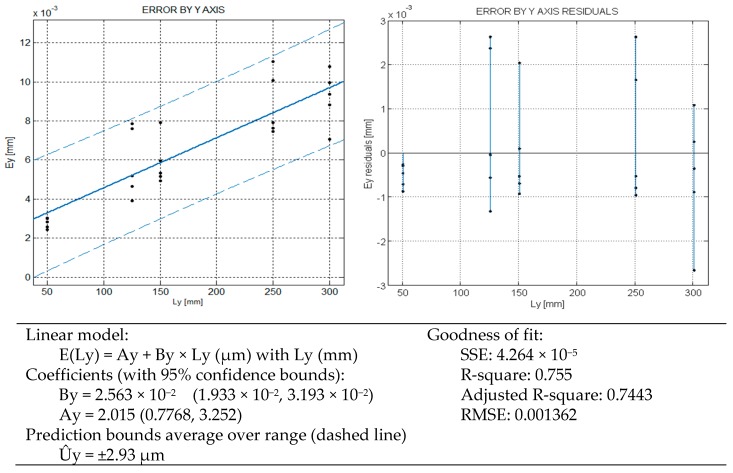
CMM error model by Y axis.

**Figure 5:**
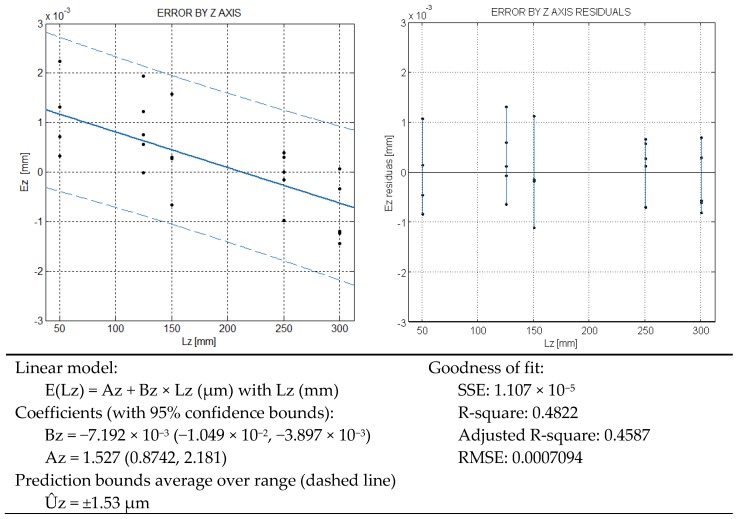
CMM error model by Z axis.

The verification, under proposed model, only checks the three axes independently. For this reason the variability or spread of the measurements has not the clear heteroscedastic appearance that in the bulk test exhibits: the residuals do not show a functional trend to grow with length, see Figure 3, Figure 4 and Figure 5. The regression by axis reflects that the mean error is approximately linear function with the length of measurement. This underlying growing spread with the length is alike because in a CMM a main source of error is the angular error of the machine [8]. These errors are captured by considering independently each axis error.

Note that the gauge blocks are grade 0 until 150 mm and grade 2 over 150 mm. Nevertheless, the length variability associated to the gauge grade difference is below the resolution of the CMM, so no evidence of blocks’ quality influence is appreciated in the experimental results.

In Figure 3, only the results for the X axis are represented. The error sum of squares SSE is lower than for the bulk model. The prediction bounds for the error are ±2.32 µm with a probability of 95%. The R-square coefficient indicates that up to 70% of the variability is explained by the regression line. It means that a new measurement by the X axis will be inside that interval around the model value at 95% level of confidence. A maximum spread or repeatability of 4.5 µm for the 300 mm block is obtained.

With the same meaning of Figure 3, in Figure 4 the results corresponding to the Y axis are represented. In this case, the linear model explains about 75% of the variability and the prediction bounds are ±2.93 µm. The maximum repeatability in the Y axis is 3.95 µm.

The results of the verification by the Z axis are represented in Figure 5. The model shows a fairly constant relationship of the average error over the Z range, compared with the X and Y axes. As a consequence, R-square is low and shows only about a 50% variability explanation by the regression line. The SSE shows a similar goodness of the model and the prediction bounds are ±1.53 µm. A maximum repeatability of 2.23 µm is obtained.

In addition, we must note that the values of adjusted R-square are close to those of R-square in the regression by axis, so the sampled points has an effective contribution to R-square. Conversely, in the bulk model the adjusted R-square is even negative, so the aggregation of all points into a bulk regression model does not contribute to the proposed regression model.

There are two methodological points about the former model. First, the bilateral measuring following the recommendations of ISO 10360 on the faces of a gauge block is properly compensated against slight misalignment of the block with the measurement axis and the five points on each face determine a least-squares plane of reference to the points of the opposite face, following recommendation of ISO 10360-2 Appendix C. This criterion of least-squares averaging the block face plane is in accordance with the reference ISO standard and its behavior has been experimentally tested versus the minimum zone tolerance alternative [9], with consistent good results. A second point of interest is the meaning of the overall prediction of error bounds for a new single observation or measurement. It will include the variability estimation by the mean square error for n − 2 degrees of freedom, but also the error caused by the variance of the slope of the regression line, amplified by the distance of the value to the centroid of the dataset and the variation of the centroid properly [10]. The formulation of the prediction bounds *P* for the regression model (E = A + Bx), with a dataset of size *n*, it is given by Equation (1), with *t* being the critical value at 95% confidence of the t-student with n − 2 degrees of freedom.

(1) $$\begin{array}{l} P=y\pm t\sqrt{\frac{SSE}{n-2}\left(1+\frac{1}{n}+\frac{L-\bar{L}}{S_{xx}}\right)};\\ \mathrm{where}\;SSE=\sum {\left(E_{i}-A-B\cdot L_{i}\right)}^{2};\;Sxx=\sum {\left(L-\bar{L}\right)}^{2};\;\bar{L}=\frac{\sum L_{i}}{n}; \end{array}$$

### 2.2. CMM Repeatability Estimation

The repeatability results expressed as the standard deviation of the results must be incorporated into the uncertainty budget [11]. Nevertheless, the ISO 10360-2 defines R_0_ as the range (maximum minus minimum value). In the case of a set of measures of non-calibrated artefacts, the mean value is not an estimation of the true value due to the presence of error. When measuring calibrated artifacts the difference between the mean value of the measurements and the assigned value to the calibrated gage can be considered a bias correction. Consequently, the standard deviation of the measurements under repeatable condition is a contribution to the uncertainty. Repeatability conditions include the same experimental hardware (CMM and gauges), the same operator, used under the same measurement operational procedure, in the same location and with repetitions over a short period of time.

**Figure 6:**
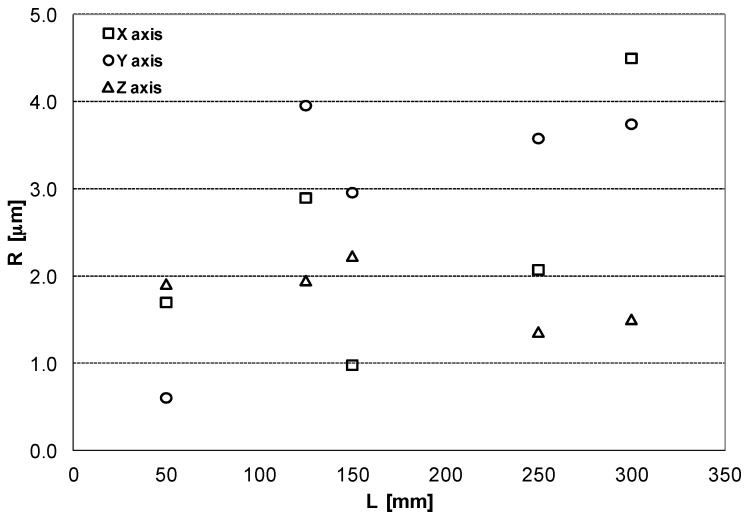
CMM repeatability R_0_ by axis.

Under the rated operating conditions of the CMM, three repetitions are specified in ISO 10360-2, just for verification purposes. Based on a such a reduced number of samples, three, a direct estimation of the standard deviation of the mean could include a big deviation [12,13]. In our test up to 50 measurements to determine 5 error values are used for each length of test, that is presumed more representative of the CMM behavior. In the verification test of Figure 3, Figure 4 and Figure 5, the maximum repeatability has been 4.5 µm and the grand average by axes and the different lengths is 2.7 µm, Figure 6.

### 2.3. CMM Probe Error Estimation

While the estimation of length error is an application to distance measurement or diameters, in general probe error must be included in the estimations of form tolerance. The verification of the maximum permissible error of the probe E_P,MPE_ is accomplished by measuring the roundness error of calibrated artefacts. According to ISO 10360-5 [14], the evaluation should be made with the radii range (maximum minus minimum) after determining the minimum least-squares sphere of the dataset from the measurement of a reference sphere. Note that the tolerance of form can be assessed by the least-squares or minimum zone tolerance criteria, and the last one is preferred by ISO 1101. Nevertheless, in general the least-squares criterion is more robust in the presence of outliers and its algorithm is widespread and easier.

According to ISO 10360-5, probe error should be verified positively before proceeding with ISO 10360-2 length verifications, and it is checked at each initial CMM setup on its own reference sphere of about 30 mm diameter. Therefore, the probe center offset and the form error in the probe is always evaluated before starting measuring operations. For the former batteries of measurements, the same probe of 4 mm nominal diameter has been used. An overall mean indicated error of sphericity of 0.004 mm has been appreciated by the CMM across probe setups.

An independent verification of the probe error will be obtained in Section 4 through a certified glass hemisphere with roundness of tens of nanometers. Even when ISO 10360-5 mentions the error of sphericity by minimum least-squares criteria, the standard is certified in its roundness, not its sphericity, thus the measurement trials will be in planes to get circles and evaluate their roundness.

## 3. Error and Uncertainty Model Overall Evaluation Results

In Part I of the model development, the relationship between the Abbé error and the slope B of error model by each axis was formulated. This linear error propagation model is valid for any length dimension, so the L matrix is singular, also evident by inspection (Equation (2)). The pseudoinverse allows getting a particular solution of the linear system. We can estimate the errors B from the error by each axis through the models of Figure 3, Figure 4 and Figure 5 for a reference length of 150 mm = Lx = Ly = Lz, and measured by the center of the volume of work by each axis. In our machine, the Abbé distances by the middle of the work volume are Dx = 225 mm, Dy = 250 mm and Dz = 50 mm, approximately. With those inputs the matrix results in Equation (2), where bold symbols denote a matrix or vector.

These values of overall angular errors in the XY plane are fully consistent with those found in the verification through the squareness of a calibrated artefact in the same machine [3]:

(2) $$\begin{array}{l} \left[\begin{array}{c} B_{x}\cdot L_{x}\\ B_{y}\cdot L_{y}\\ B_{z}\cdot L_{z} \end{array}\right]=\left[\begin{array}{ccc} 0 & -D_{y} & D_{z}\\ D_{x} & 0 & -D_{z}\\ -D_{x} & D_{y} & 0 \end{array}\right]\left[\begin{array}{c} \theta_{x}\\ \theta_{y}\\ \theta_{z} \end{array}\right];\;B=D\cdot \theta ;\\ \left[\begin{array}{c} -2.7225\\ 3.8445\\ -1.0788 \end{array}\right][\mu m]=\left[\begin{array}{ccc} 0 & -250 & 50\\ 225 & 0 & -50\\ -225 & 250 & 0 \end{array}\right]\;\cdot 10^{3}[\mu m]\cdot \left[\begin{array}{c} \theta_{x}\\ \theta_{y}\\ \theta_{z} \end{array}\right]\to \left[\begin{array}{c} \theta_{x}\\ \theta_{y}\\ \theta_{z} \end{array}\right]=\left[\begin{array}{c} 0.1580\\ 0.0985\\ -0.0548 \end{array}\right]\cdot 10^{-4}[\mathrm{rad}]=\left[\begin{array}{c} 3.25\\ 2.03\\ -1.13 \end{array}\right][\prime \prime ] \end{array}$$

After considering the average explained variability of the linear model, the non-explained variability in the regression model can be incorporated into the uncertainty budget. This approach is used when the overall estimation E_L,MPE_ is taken as a first estimation of the machine uncertainty [11] in those testing conditions. In the case of our proposed model the non-explained uncertainty is sensibly lower. For a proper estimation of the uncertainty in other valid operative conditions of the CMM operational range, all other factors that introduce variability should vary in the experimentation [15,16]. Considering the variability of the error a random variable, and due to the real interdependence of CMM hardware in the error of the coordinates, we can expect covariance in the error between axes, so four out of the seven testing positions in the ISO bulk model are diagonal directions in the cube of the volume of measurement. Because the proposed model only evaluates variances by axis, the issue is whether the variability of error is infra-evaluated or not.

Two supporting facts of the consistency of the model can be pointed out: As already mentioned, the overall maximum permissible error by the ISO model (seven trial directions) and the proposed model are very similar, about ±6.7 µm. The second fact is the result of the regression. Most of the total variability is explained by axis and it is captured by the proposed model. Note that the SSE in the bulk model is 8 × 10^−4^, while the SSE sum from the three different axes is 8 × 10^−5^, so a big portion of the non-explained variability present in the bulk model or the ISO model is explained through the proposed model by axis, reducing of an order of magnitude the sum square error, with little residual covariance.

As already mentioned, the verification of the CMM following ISO 10360 verification tests produces an overall error quantification very similar to the error model presented just by evaluating the three main axes. In this sense the proposed model by axis seems to capture the variability of error of the CMM. Next, an additional trial on the XY plane is accomplished to measure directly the errors and compare the experimental result with the predicted values of the proposed model. The measurement of a gage block of nominal 175 mm is checked, oriented in a direction of 45° with respect to Z and the projection on the XY plane is 30° with respect to X. The direct estimation of the standard uncertainty u is given by Equation (3), where the estimation of the variance is based for small samples on the t-distribution with n − 1 degrees of freedom.

(3) $$\begin{array}{l} \mathrm{Set}\;\mathrm{of}\;\mathrm{measurements}.\;\mathrm{CMM}\;\mathrm{Indication}\;[\mathrm{mm}]\\ x_{i}=(174.99681,\;175.00248,\;175.00183,\;174.99931,\;175.00067);\;n=5\\ \bar{x}=175.00022\;\mathrm{mm};\;s=\sqrt{\frac{{\left(x_{i}-\bar{x}\right)}^{2}}{n-1}}=2.256\;\mu m;\;u=\sqrt{\frac{n-1}{n-3}}\frac{s}{\sqrt{n}}\;=1.43\;\mu m \end{array}$$

Considering the uncertainty by axis estimated by the proposed model, the standard uncertainty by the law of propagation of uncertainty can be estimated, under the initial hypothesis of independent variables, see Part I of this work, by Equation (4).

Noteworthy, the uncertainty model resulting from the error model of length does not depend on the length itself in a first order approach. This means that the uncertainty does not grow with the length by axis in a first order approach. This has a correspondence with the homocedastic behavior of error in Figure 3, Figure 4 and Figure 5, where the error variability is approximately constant. Note that the independence of x1, x2, y1, y2, z1, z2 could be alike when only one axis varies at a time. The independence of x1 and x2, for instance, is also supported by following good practices of bilateral measurement, according to the recommendations of ISO 10360-2.

(4) $$\begin{array}{l} L=175\;\mathrm{mm};\;\varphi =45{}^{\circ};\;\theta =30{}^{\circ}\to \cos\varphi =\sin\varphi =\frac{1}{\sqrt{2}};\;\sin\theta =\frac{1}{2};\;\cos\theta =\frac{\sqrt{3}}{2}\\ \mathrm{From}\;\mathrm{the}\;\mathrm{error}\;\mathrm{models}\;\mathrm{Fig}\;X-Y\;,\;\mathrm{the}\;\mathrm{expanded}\;\mathrm{uncertainty}\;(\mathrm{coberture}\;\mathrm{coefficient}\;k=2)[\mu m]\text{:}\\ {\hat{U}}_{x}=2.32;\;{\hat{U}}_{y}=2.93;\;{\hat{U}}_{z}=1.53\\ \mathrm{Thus},\;\mathrm{the}\;\mathrm{standard}\;\mathrm{uncertainties}\;u_{x}=1.16;\;u_{y}=1.47;\;u_{z}=0.77\\ u^{2}=2{\left(\cos\varphi \cos\theta \right)}^{2}\cdot {u_{x}}^{2}+2{\left(\cos\varphi \sin\theta \right)}^{2}\cdot {u_{y}}^{2}+2\sin^{2}\varphi \cdot {u_{z}}^{2}=1.01+5.37+5.85=2.13\;\mu m^{2};\\ u=1.46\;\mu m \end{array}$$

The uncertainty obtained by the direct estimation from the sample of measurements (Equation (3)) and that obtained from the proposed model of independent variable and standard uncertainty by axis (Equation (4)) can be compared. The results are the same value, *u* = 1.4 µm. The proposed model captures most of the error variability (uncertainty), so the covariance can be disregarded in a first order approach. Further experimental evidence should corroborate these results following the methodology. The error model by axis based on length measurement captures the trend of the errors and the remaining non-explained or random variability becomes uncertainty, and it is uniform by each axis in a first order approach for any length.

## 4. Experimental Feature Results and Discussion

### 4.1. Rectangular Gauge Blocks

Case 1 is a calibrated gauge block of nominal length 175 mm measured with azimuth angle with respect to the Z axis, and with respect to the X axis. A proper orientation of the block axis is corrected properly by the normal to the faces through the measurement process. It is measured 50 times, from five repetitions in a bilateral technique with 10 points sampled, five on each block face. The results are compared to those expressed by the ordinary model of E_L,MPE_. In addition, Case 2 is a calibrated gauge block of 300 mm lying on the granite table with its axis oriented with respect to X. It is measured five times on five points on the two faces, so a total of 50 times, either way. The results for both cases are listed in Table 1.

In Case 1, there is no remarkable difference between models in the best value of measurement after correction. The proposed model and the ISO model pass by the centroid of the dataset and the block of 175 mm is by the centre of the range of verification (50 ÷ 300 mm). Nevertheless, the estimation of uncertainty is smaller by our proposed model with respect the E_L,MPE_. In Case 2, a block of 300 mm by the extreme of the range of verification is used. The proposed model presents a better result of the measured value, more accurate and precise than if the standard ISO model was applied.

### 4.2. Flatness of the CMM Granite

A sample of 30 points is taken from the granite of the CMM by the center of the measurement volume, Table A1. The calculation of flatness by an accurate algorithm [17] results in a normal direction of the surface by the Z axis of the CMM and it identifies the critical points of the sampled surface, Table 2.

From the calculation of the four configurations a flatness value of 0.002 mm after indication correction with the mean error is obtained. A conservative uncertainty estimation can be the maximum value from the four configurations, 0.00283 mm. In the proposed model the uncertainty from error variability (Equation (5)) is estimated. Additionally, the uncertainty of flatness from the projection of the vectorial point uncertainty in the direction of the surface [14] can also be directly estimated. Note the basic agreement for the sample between the proposed method (Equation (5)) and the alternative (Equation (6)):

(5) $$U_{k=2}=2\sqrt{\frac{c-1}{c-3}}\frac{s}{\sqrt{c}};\;s=\sqrt{\frac{{\left(E_{i}-\bar{E}\right)}^{2}}{c-1}\;}\to \;U_{k=2}=0.00216\;\mathrm{mm}$$

(6) $$U_{k=2}=\sqrt{2}\cdot \hat{U}\cdot n=\sqrt{2\cdot }(0.00232,\;0.00293,\;0.00153)\cdot (0,0,1)=0.00216\;\mathrm{mm}$$

From the former calculations a flatness measurement of 0.002 ± 0.00216 mm can be offered. Note that the granite is flat about 0.002 from indication, but applying the proposed model an estimation of uncertainty can be provided. The uncertainty and flatness are in fact at the same level of the mean repeatability of the machine, estimated in Section 2 of about 0.003 mm. Therefore, we can infer that the granite is flat at the level of the CMM repeatability.

### 4.3. Angle Blocks

Angle blocks are realization of an angle defined by two faces. Regardless of the theoretical consideration about the unit of measurement (radian or dimensionless) [18] the angle blocks can be evaluated from the proposed model of direct vectorial calculation incorporating the errors and the uncertainty of the distance between the critical points that define the each face of the angular block. Therefore, the dihedral angle model and the minimum zone criteria are used.

**Figure 7:**
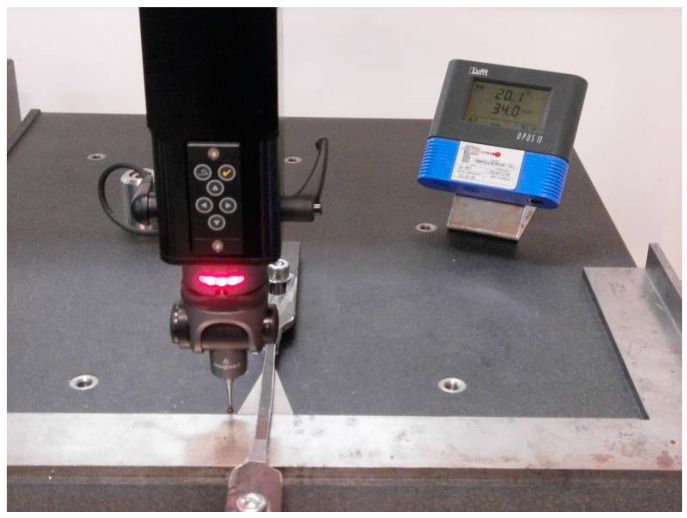
Angle gauge block measuring.

An angle block calibrated with assigned value in the interval 45° ± 2″ is measured with its axis aligned with the Y axis of the CMM, Figure 7, with CMM coordinate indication in Table A2. The uncertainty of the block is beyond of the resolution of the CMM. Note that the resolution of the CMM 0.001 mm is equivalent for a block face of 50 mm long to the resolution of 2.10–5 rad or 4″. In addition, the flatness of the faces has been verified through the CMM and the indication is not less than 0.004 mm in the datasets, equivalent to 16″ on each face. This gives an order of magnitude of the smaller angle that could be confidently resolved by the CMM.

Like in the rectangular gauge blocks, the probe error is not considered in the error budget, because the probe contact point is almost the same when measuring each face. The comparison between the indication by the least-squares method and the minimum zone method shows a closer result to the assign value by the least-squares method before any correction, Table 3. After correction, the proposed model presents a better result, but more importantly, it provides an uncertainty estimation. The final result is very close to the assigned value of the standard. The uncertainty interval at the order of minute is consequence of the resolution of the machine and the measured flatness of the block gauges.

It must be considered that the error regression interval has been established over a range from 50 to 300 mm in Section 2, so the CMM error compensation at the lower bound is thus being used. Also remarkable is the good behavior of the MZ model even when the angular blocks are ordinarily calibrated by the least-squares method. Even when the minimum zone tolerance evaluates better the flatness of surfaces as the geometrical substitution of the physical surface, the gauge blocks are calibrated using the least-squares method, so a formerly proper agreement is expected between the indication and the assigned value of the standards through the least-squares method [9]. This is the behavior in Case 4, when calculating from indication directly. Nevertheless, the minimum zone is more effective when the error correction is applied, with the advantage of giving an uncertainty estimation. Because of a sample of size 3, the estimation based on the t-student distribution is not feasible. A conservative estimation of uncertainty can be the maximum reached across the three samples. Another alternative for very small samples is to estimate uncertainty from the Craig model [13]. Its result (Equation (7)) is not very different from the estimation carried out directly through the model:

(7) $$\begin{array}{l} \mathrm{Craig}\;\mathrm{model}:\;u^{2}=\frac{m-1}{2}{\left(\frac{\Gamma \left(\frac{m-1}{2}\right)}{\Gamma \left(\frac{m}{2}\right)}\right)}^{2}s^{2};\;s=\sqrt{\frac{{\left(E_{i}-\bar{E}\right)}^{2}}{m-1}\;}=1.71\prime \\ \mathrm{For}\;m=3\;u^{2}={\left(\frac{\Gamma \left(1\right)}{\Gamma \left(\frac{3}{2}\right)}\right)}^{2}s^{2}={\left(\frac{1}{0.8862}\right)}^{2}s^{2}\to u=1.93\;\prime \to U_{k=2}=2\cdot 1.93\prime =3.86\prime =3\prime 52\prime \prime \end{array}$$

### 4.4. Glass Hemisphere

**Figure 8:**
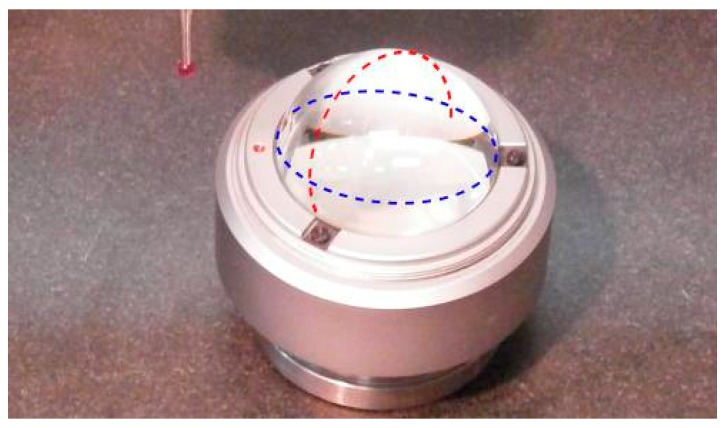
Measuring a glass hemisphere in two planes.

A glass hemisphere by Taylor Hobson with roundness error of tens of nanometer is measured in two planes, Figure 8. Its roundness error is below the resolution of the CMM, so the difference from a perfectly round indication should be assigned to the CMM’s overall error, uncertainty or the use of an approximate roundness algorithm. The two set of data are included in Table A3 and Table A4. The accurate calculation of roundness from indication is accomplished by the least-squares method based on the well-known algorithm Levenberg-Marquardt and by the accurate minimum zone method [19].

The roundness results show the error by the CMM and the probe (about 0.004 mm). Using the ISO model the roundness results from an indication cannot be adjusted. The bounds E_L,MPE_ = 6.7 µm cannot be applied. The maximum repeatability of the CMM 0.0045 mm or even the average of 0.003 mm can hardly bound a nominal roundness in the order of 0.005 mm.

The use of the proposed model allows correcting the calculated roundness from an indication. The maximum uncertainty calculated by the method can be adopted as a conservative measure of the uncertainty of the error. In the two cases of application, Case 5 in Table 4 and Table 5, Case 6 in Table 6 and Table 7, respectively, the uncertainty of the error and the maximum uncertainty estimated through the model are of the same order of magnitude, 1.96 and 1.47 μm. Case 5 represents a full circle with more sampled points, but Case 6 only measures a half of a circle on the hemisphere.

**Figure 9:**
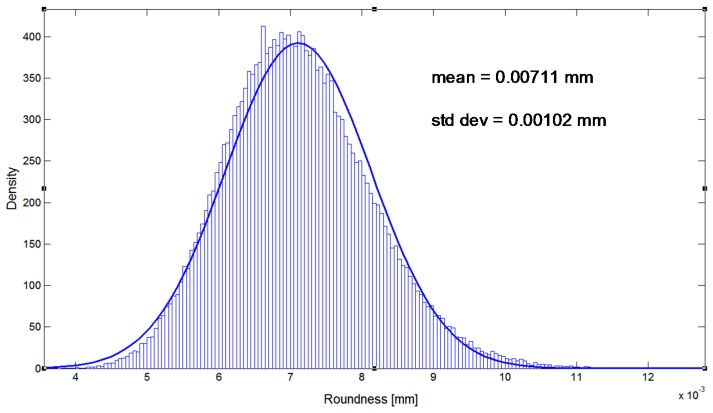
Uncertainty estimation by Monte Carlo method of Case 5.

Finally, the uncertainty of the full circle of Case 5 is estimated by the Monte Carlo method based on 100,000 shots. Considering the expanded uncertainty from our model Û(k = 2) = (0.00232,0.00293,0.00153) mm, the standard uncertainty can be obtained through u = (u_x_,u_y_,u_z_) = (0.00116,0.001465,0.000765) mm. Using the Gaussian distributions N(0,u_x_) and N(0,u_y_), the results of Figure 9 are obtained. Note that U(k = 2) = 0.00204 mm is very close to the maximum value obtained for direct estimation of the model U(k = 2) = 0.00196 mm. The order of magnitude of the estimation from the variability of the error, just from the four configurations of the solution, is U (k = 2) = 0.00147. It must be noted that the Monte Carlo mean roundness is calculated directly from indication and gives a result of 0.007 mm, higher than the 0.005 mm of the dataset of Case 5. This was observed before in [19]. It is associated with the tolerance of form as distribution of positive value and the indirect measurement of roundness obtained through a process of minimization. Nevertheless, the Monte Carlo simulation of the roundness does not consider any error correction in the indication. Because the hemisphere is free of roundness error at the resolution of the CMM, the roundness results are mainly associated to the CMM repeatability of about 2.7 µm and a probe tip error of form of about 4 µm. As a consequence, the result from just one sample based on the proposed model (Case 5) that includes error correction is 5 ± 1.5 µm. It can be compared with the result from the Monte Carlo simulation without any correction in the indication of 7 ± 2 µm. Noteworthy, the reference verification values of the CMM, maximum permissible error E_L,MPE_ = 6.7 µm or the maximum repeatability of R_0_ = 4.5 µm, can serve little to a useful expression of the roundness measurement.

## 5. Conclusions

The experimental extraction of the error model by axis has been demonstrated feasible and useful. The theoretical basis of the vectorial models of error propagation developed in Part I are endorsed by the CMM experimental behavior.

The work developed suggests the recommendation of conducting CMM verification by following standard ISO techniques, but with more repetitions than the standard three times. Up to five repetitions are used in this work, but no less than four is recommended. This can allow extracting errors by axis with a reasonable number of data for error regression. In addition, it would allow a direct estimation of uncertainty from the variability of the error.

The homocedastic behavior of the non-explained variability of error around the linear trend is a main advantage versus the conventional CMM ISO model of maximum permissible error. The model by-axis has shown the capability of capturing most of the variability of the error or the measurement. As a consequence, the non-explained variability of the measurement or the error becomes a contribution to the overall estimation uncertainty. It is a direct calculation from the machine error model by axis and through a proper vectorial composition in each measurement feature model.

The models of feature measurement based on coordinates under minimum zone tolerance criteria present over-determination in terms of error and uncertainty in some cases. For instance, flatness or roundness under minimum zone criteria are determined by four points. Those models give a unique solution, but allowing multiple estimations of the error and uncertainty based on the same dataset. This is an advantage more than a drawback towards an economy in CMM sampling. In the case of simple length measurement or an angle on a plane, the best value solution, error and uncertainty estimation, all three are unique through the proposed model.

The least-squares fittings are dominant in metrology for its robustness against outliers and widespread algorithms, but they do not determine the critical points of the form tolerance. Therefore, we can hardly profit from the analysis of errors based on the direct propagation of error and uncertainty associated with the coordinate of those critical points. The proposed model of error and the derived feature models could be approximately applied under least-squares fitting algorithms considering the points closer to the feature under definition.

The estimation of uncertainty was discussed in the development of the models in Part I and it has shown to be useful in the experimental trials in comparison with alternative estimations, in particular versus the ordinary Monte Carlo method that requires to fix the distribution of uncertainty of the points and an intense calculation in order to give just the uncertainty of one measurement. The proposed model of error by axis is capable of getting the ordinate at the origin Ai or the error for each i-axis. This can be adopted as an estimation of the point error of vectorial character, useful as an input to the Monte Carlo method.

The proposed model of error compensation makes use of the basic techniques of the standard model of maximum permissible error based on a first order approach of length errors on the zone of interest inside the volume of measurement. It is presented as an affordable alternative to a detailed mapping of coordinate errors. As regards big fields of measurement or just a quasi-linear CMM behavior, a first attempt could be the use of regression models by ranges, in order to cover properly the volume of measurement. Extensive experimental trials and results comparison should reinforce the utility of the proposed model and its potential use in laboratories or industrial environment.

## Figures and Tables

**Table 1:** Length tests from calibrated artefacts.

CASE 1	Gauge block nominal 175 mm with certified calibrated length 175.00120 ± 0.00022 mm (expanded uncertainty k = 2).		
	Mean indication L= 175.00022 mm (without CMM bias correction).		
	Position: ϕ = 45°; θ = 30°		
**Model ISO**			**Proposed Model**
L_m_ = L + bias ± E_L,MPE_ = L_nm_ ± E_L,MPE_			L_m_ = L + E ± Û = L_nm_ ± Û
Bias = 2.95 µm (by Figure 2)			L_nm_ = 175.00442 mm
E_L,MPE_ = 6.74 µm (by Figure 2)			E = L_nm_− L = 4.20 µm
L_nm_ = L + bias = 175.00022 + 0.00295 = 175.00317 mm			Û = 3.71 µm
L_m_ = 175.003 ± 0.0067 mm			L_m_ = 175.004 ± 0.0037 mm
CASE 2	Gauge block nominal 300 mm with certified calibrated length 299.99939 ± 0.00026 mm (expanded uncertainty k = 2).		
	Mean indication L = 299.99402 mm (without CMM bias correction)		
	Position: ϕ = 0° ; θ = 30°		
**Model ISO**			**Proposed Model**
L_m_ = L + bias ± E_L,MPE_ = L_nm_ ± E_L,MPE_			L_m_ = L + E ± Û = L_nm_ ± Û
Bias = 2.95 µm (by Figure 2)			L_nm_ = 300.00019 mm
E_L,MPE_ = 6.74 µm (by Figure 2)			E = L_nm_− L = 0.00617 mm
L_nm_ = L + bias = 399.99402 + 0.00295 = 399.99697 mm			Û = 0.00370 mm
L_m_ = 299.997 ± 0.0067 mm			L_m_ = 300.000 ± 0.0037 mm

**Table 2:** Granite flatness measurement. CASE 3. Critical points (2,4–3,20), Data Table A1, Values in (mm).

	u	v	t	MZF_uvt_	E_uvt_	Û_uvt_
C1	(−0.183,23.678,0)	(−153.594,101.860,0)	(13.753,13.039,0.002)	0.0019995	0.0002295	0.00130
C2	(−0.183,23.678,0)	(−153.594,101.860,0)	(−139.841,114.899,0.002)	0.0019995	−0.0012973	0.00283
C3	(−0.183,23.678,0)	(−153.594,101.860,0)	(13.936,−10.639,0.002)	0.0019995	0.0017561	0.00023
C4	(−0.183,23.678,0)	(−153.594,101.860,0)	(−139.658,91.221,0.002)	0.0019995	0.0002294	0.00130
			Mean	0.002	0.00023	

**Table 3:** Angle block measurement results. CASE 4. Angle gauge block 45° ± 2″. Data Table A2.

Dataset #	Critical Points RF	Critical Points LF	Angle from Indication LS α	Angle from Indication MZ α	Error Eα	Angle α_nm_	Uncertainty ± Uα
1	(7,3–8,10)	(8,5–6,1)	44°59′37″	45°3′38″	−3′48″	44°59′40″	±3′40″
2	(3,10–2,7)	(8,1–10,7)	45°0′7″	45°3′44″	−3′43″	45°0′1″	±3′14″
3	(8,1–5,10)	(10,3–9,6)	45°0′24″	45°0′53″	−0′48″	45°0′6″	±0′16″
Measurement result			45°0′9″	45°2′45″	−2′30″	44°59′55″	±3′40″

**Table 4:** Roundness measurement results of Case 5. Roundness from dataset Table A3. Values in (mm).

	MZR	R	a	b
Minimum Zone	0.00522	26.03354	232.02654	253.39571
Least squares	0.00531	26.03335	232.02664	253.39580

**Table 5:** Roundness measurement Case 5. Error and uncertainty results for minimum zone criteria. Values in (mm).

Critical Points			Error and Uncertainty				
Point #	x (mm)	y (mm)	Critical points	8,15	21,29	8,29	21,15
8	222.871	277.769	E (mm)	−0.000046	−0.001841	−0.000147	0.000844
21	256.453	244.383	U [mm] (k = 2)	0.000130	0.001960	0.000200	0.000736
29	226.953	227.864	Mean value E [mm]	−0.00030			
15	251.574	270.586	Max value U [mm]	0.00196			
			Uncertainty of E (n = 4; k = 2)	0.0015			
			$s=\sqrt{\frac{{\left(E_{i}-\bar{E}\right)}^{2}}{n-1}};u=\sqrt{\frac{n-1}{n-3}}\frac{s}{\sqrt{n}};U=k\cdot u$				
			MZR_m_ = MZR + E ± Û = 0.00522 − 0.00030 ± 0.00196 = **0.005 ± 0.00196 mm**				

**Table 6:** Roundness measurement results Case 6. Roundness from dataset Table A4. Values in (mm).

	MZR	R	a	b
Minimum Zone	0.00568	25.36138	253.39556	−556.72247
Least squares	0.00712	25.35867	253.39569	−556.71841

**Table 7:** Roundness measurement Case 6. Error and uncertainty results for minimum zone criteria. Values in (mm).

Critical Points			Error and Uncertainty				
Point #	x (mm)	y (mm)	Critical points	2,8	2,24	15,8	15,24
2	228.701	-430.499	E (mm)	0.000246	−0.000036	−0.000233	−0.000515
15	261.699	−414.988	U [mm] (k = 2)	0.000016	0.000003	0.000011	0.000030
24	279.314	−431.132	Mean value E [mm]	−0.00013			
8	235.451	−420.992	Max value U [mm]	0.00003			
			Uncertainty of E (n = 4; k = 2)	0.00002			
			$s=\sqrt{\frac{{\left(E_{i}-\bar{E}\right)}^{2}}{n-1}};u=\sqrt{\frac{n-1}{n-3}}\frac{s}{\sqrt{n}};U=k\cdot u$				
			MZR_m_ = MZR + E ± Û = 0.00568 − 0.00013 ± 0.00003 = **0.006 ± 0.00003 mm**				

## Appendix A

**Table A1:** Granite flatness.

Point #	x [mm]	y [mm]	z [mm]
1	150.569	161.800	−485.069
2	151.136	138.495	−485.068
3	137.383	125.456	−485.070
4	151.319	114.817	−485.068
5	138.866	103.311	−485.069
6	150.303	95.111	−485.068
7	137.885	84.617	−485.069
8	151.491	72.219	−485.068
9	141.419	64.847	−485.069
10	206.130	218.320	−485.070
11	186.961	202.538	−485.070
12	207.748	190.807	−485.069
13	189.576	130.952	−485.070
14	192.284	94.076	−485.070
15	247.065	194.149	−485.068
16	251.169	173.446	−485.069
17	264.555	118.727	−485.069
18	250.302	84.049	−485.068
19	266.562	75.570	−485.068
20	265.033	59.649	−485.070
21	249.690	44.504	−485.070
22	263.665	40.053	−485.068
23	249.230	25.474	−485.069
24	289.791	74.976	−485.070
25	305.326	29.075	−485.069
26	291.529	112.159	−485.068
27	305.250	103.548	−485.068
28	291.550	62.152	−485.070
29	310.033	35.141	−485.068
30	290.977	23.596	−485.070

**Table A2:** Angle block datasets.

		LEFT FACE (LF)			RIGHT FACE (RF)		
Dataset #	Point #	x [mm]	y [mm]	z [mm]	x [mm]	y [mm]	z [mm]
1	1	223.391	131.940	−479.883	254.591	91.711	−479.617
	2	219.711	122.976	−479.884	250.055	102.574	−479.616
	3	215.284	112.192	−479.884	246.235	111.721	−479.616
	4	210.959	101.660	−479.884	241.530	122.981	−479.616
	5	207.223	92.559	−479.885	237.607	132.371	−479.616
	6	207.222	92.569	−484.329	237.602	132.374	−484.561
	7	211.098	101.999	−484.329	242.478	120.712	−484.562
	8	216.158	114.316	−484.328	246.361	111.405	−484.561
	9	219.984	123.640	−484.328	249.718	103.376	−484.562
	10	223.168	131.386	−484.328	253.548	94.212	−484.562
2	1	223.169	131.388	−480.184	254.292	92.425	−480.554
	2	217.718	118.119	−480.185	250.579	101.327	−480.554
	3	215.051	111.622	−480.185	246.421	111.260	−480.553
	4	210.872	101.451	−480.185	242.201	121.368	−480.554
	5	207.353	92.879	−480.185	238.628	129.927	−480.554
	6	207.352	92.881	−484.349	238.623	129.933	−484.407
	7	211.612	103.250	−484.349	242.358	121.002	−484.408
	8	214.910	111.289	−484.349	245.694	113.009	−484.408
	9	218.950	121.122	−484.348	249.929	102.875	−484.408
	10	223.357	131.840	−484.348	254.222	92.588	−484.408
3	1	223.500	132.196	−480.200	254.303	92.395	−480.549
	2	218.929	121.062	−480.200	250.952	100.421	−480.548
	3	215.763	113.362	−480.200	245.875	112.576	−480.548
	4	210.111	99.592	−480.201	241.529	122.983	−480.548
	5	207.042	92.123	−480.201	238.649	129.877	−480.548
	6	207.035	92.111	−485.026	238.649	129.872	−484.622
	7	210.512	100.568	−485.026	242.328	121.055	−484.622
	8	215.147	111.856	−485.025	246.452	111.184	−484.623
	9	219.143	121.583	−485.025	251.199	99.834	−484.622
	10	223.352	131.843	−485.025	254.316	92.407	−484.623

**Table A3:** Hemisphere datasets. (a) Plane Z constant.

Point #	x [mm]	y [mm]
1	205.976	254.255
2	206.318	258.106
3	207.360	262.263
4	209.214	266.456
5	211.945	270.467
6	214.987	273.581
7	217.545	275.526
8	222.852	278.254
9	227.663	279.547
10	231.866	279.914
11	237.052	279.422
12	239.983	278.663
13	244.596	276.667
14	248.560	273.976
15	251.555	271.071
16	255.067	265.963
17	256.590	262.452
18	257.689	258.139
19	258.013	252.699
20	257.509	248.634
21	256.434	244.868
22	254.394	240.594
23	251.271	236.368
24	247.770	233.160
25	243.252	230.401
26	238.871	228.770
27	236.053	228.164
28	230.943	227.869
29	226.934	228.349
30	221.842	229.914

**Table A4:** Hemisphere datasets. (b) Plane X constant.

Point #	y [mm]	z [mm]
1	228.089	−432.084
2	228.701	−430.499
3	229.423	−428.951
4	230.208	−427.495
5	231.142	−425.993
6	232.255	−424.450
7	233.343	−423.134
8	235.451	−420.992
9	237.200	−419.522
10	239.410	−417.991
11	242.458	−416.348
12	246.687	−414.810
13	253.367	−413.853
14	258.186	−414.191
15	261.699	−414.988
16	265.688	−416.526
17	268.427	−418.036
18	271.400	−420.195
19	273.053	−421.691
20	274.557	−423.293
21	276.085	−425.224
22	277.075	−426.699
23	278.343	−428.955
24	279.314	−431.132
